# Contour angle and peri‐implant tissue height: Two interrelated features of the implant supracrestal complex

**DOI:** 10.1002/cre2.731

**Published:** 2023-03-29

**Authors:** Algirdas Puisys, Martin Janda, Viktorija Auzbikaviciute, German O. Gallucci, Nikos Mattheos

**Affiliations:** ^1^ VIC Clinic, Private Practice Vilnius Lithuania; ^2^ Department of Prosthodontics, Faculty of Odontology Malmoe University Malmö Sweden; ^3^ Department of Restorative Dentistry and Biomaterials Sciences School of Dental Medicine Harvard University Boston Massachusetts USA; ^4^ Department of Oral and Maxillofacial Surgery, Faculty of Dentistry Chulalongkorn University Bangkok Thailand; ^5^ Department of Dental Medicine Karolinska Institute Stockholm Sweden

**Keywords:** emergence angle, implant prosthesis contour, implant supracrestal complex, peri‐implant tissue height

## Abstract

**Objectives:**

Recent research has suggested the contour of the prosthesis and the vertical height of the peri‐implant mucosa as important parameters that can influence the long term health and stability of the peri‐implant tissue. In particular, overcontouring of the prosthesis has been correlated with an increased risk for peri‐implantitis, while reduced soft tissue height has been associated with marginal bone loss, recession, and other soft tissue complications. Although these two parameters have been investigated as independent in the current literature, clinical experience points toward a close interrelation between transmucosal tissue height and prosthesis contour angle. It is often found that a reduced vertical height of the implant supracrestal complex is the main reason for overcontouring of the prosthesis. At the same time, achieving a favorable contour of 30o or less is not possible unless the clinician has ensured an adequate vertical height of the soft tissue. The purpose of this short communication is to establish the relation between tissue vertical height and prosthesis contour by utilizing a theoretical geometry equation based on the Pythagorean theorem. In doing so, one can use the dimensions of the implant as well as those of the prosthesis at the mucosal margin to calculate the essential vertical height for achieving a favorable prosthesis contour.

**Conclusions:**

As the treatment plan of the implant supracrestal complex is “top‐down,” in case of deficient vertical height, subcrestal placement of the implant should be considered to achieve a proper prosthesis contour.

## 1 INTRODUCTION

An increasing body of evidence points toward significant interrelations between the design of the implant prosthetic elements (Mattheos, Vergoullis, et al., 2021) and the health of the peri‐implant tissue. The contour of the prosthesis and the dimensions of the peri‐implant mucosa are the features most frequently implicated in the long term health and stability of the peri‐implant tissue (Mattheos, Janda, et al., 2021).

Three cross‐sectional studies based on peri‐apical radiographs (Katafuchi et al., 2018; Majzoub et al., 2021; Yi et al., 2020) have suggested that overcontouring of the prosthesis of more than 30° is correlated with an increased risk for peri‐implantitis in bone‐level implants. Although two cross‐sectional studies that have followed a similar methodology have not confirmed the correlation of overcontouring with peri‐implant inflammation (Inoue et al., 2020; Lops et al., 2022), these results overall certainly warrant further research exploration. Aside from peri implantitis, animal (Souza et al., 2018) and clinical (Spinato et al., 2019) studies have shown that a wide angle of the profile of the abutment as it is ascending from the bone‐level can lead to early marginal bone loss. Furthermore, the convexity of the prosthesis profile has been correlated with increased recession (Siegenthaler et al., 2022), marginal bone loss (Valente et al., 2020), and when combined with overcontouring, Peri‐implantitis (Yi et al., 2020).

The peri‐implant soft tissue dimensions and, in particular, the vertical “height” of the mucosa have also been shown to be important parameters for health and stability. The initial concepts of the peri‐implant mucosa height were influenced by concepts first described in the periodontium, from the “Biologic Width” (Gargiulo et al., 1961) to the recently introduced “Supracrestal Tissue Attachment” (Caton, et al., 2018). Terms such as “Peri‐Implant Soft Tissue Barrier” (Glauser et al., 2005), “Peri‐implant Mucosa” (Araujo & Lindhe, 2018), and “Peri‐implant Phenotype” (Avila‐Ortiz et al., 2020) have been utilized to describe the peri‐implant tissues. These studies have advocated the importance of an essential minimum height of the peri‐implant mucosa to ensure the stability of the soft tissue and marginal bone in the long term. This essential height, which has previously corresponded to the concept of the “Biologic width,” has been approximated to be between 2.5 and 4 mm in human studies (Glauser et al., 2005; Romanos et al., 2010; Tomasi et al., 2014). Failure to secure this soft tissue height has been associated with marginal bone loss (Linkevicius, Puisys, Linkevieiene, et al., 2015; Linkevicius, Puisys, Steigmann, et al., 2015), recession, and other soft tissue complications.

The recently introduced concept of the Implant Supracrestal Complex (Mattheos, Vergoullis, et al., 2021) suggested that the dimensions and morphology of the peri‐implant mucosa are interrelated with the design of the prosthesis, from which it cannot be studied in isolation. Understanding these interrelations, as well as the clinical implications of the design of the prosthesis, the transmucosal components, and implant position can help devise effective implant treatments and reduce the risk of complications in the long term.

The aim of this short communication is to discuss the interrelation between the prosthesis contour and the vertical dimension of the peri‐implant mucosa, further suggesting a mathematical equation to support clinically relevant guidelines for the design of the Implant Supracrestal Complex.

## 2 PERI‐IMPLANT TISSUE HEIGHT, “BIOLOGIC WIDTH,” AND CLINICAL IMPLICATIONS

The micromorphological structure and dimensions of the peri‐implant mucosa have been the focus of research since the early 1990s. The first studies were conducted by means of histomorphometry in animals, which offered some important observations with regard to the biological structure of the peri‐implant tissue. From apical to coronal in the vertical direction, the peri‐implant tissues are defined by the Marginal Bone (MB), the Connective Tissue (CT), the Junctional Epithelium (JE), and the Sulcus (S) lined with sulcular epithelium. CT in implants appears with no vascular supply close to the abutment and very few fibroblasts, resembling more scar tissue; this is likely attributed to a lack of the PDL vascular complex (Glauser et al., 2005). Blood vessels originating from the supra‐periosteal complex are located in the lateral borders of the CT and JE zone. These blood vessels are the origin of the immune response to bacteria in the sulcus (Berglundh et al., 1994; Kawahara et al., 1998). Similar to teeth, peri‐implant crevicular fluid is produced, which flows into the sulcus through the junctional epithelium. Analysis of peri‐implant crevicular fluid (PICF) for protein biomarkers such as pro‐inflammatory cytokines, chemokines, and bone turnover markers can reveal clinical and subclinical inflammation (Güncü et al., 2012).

Apart from structural observations, animal studies were used to identify the dimensions of the peri‐implant mucosa, especially in comparison to natural teeth (Table 1). The very diverse anatomic conditions and sizes, however, on the different animal models, combined with the different conditions of implant placement (e.g., absence of mucosal scalloping, diverse sulcus depth, no esthetic needs), prevent extrapolation of any conclusions on peri‐implant tissue dimensions relevant to humans.

**Table 1:** Calculation of the peri‐implant tissue vertical dimensions in animal histomorphometric studies.

Author/year	Animal	Tissue vert. dimension (range in mm)
Quaranta et al. (2008)	Monkey	3.4–5.3
Todescan et al. (2002)	Mongrel	2.8–4.3
Berglundh et al. (1991)	Beagle	3.80
Hermann et al. (2001)	Foxhound	2.8–3.5
Baffone et al. (2015)	Labrador	2.5–2.8
Farronato et al. (2012)	Minipigs	1.9–3.2

The few human studies available have pointed to significant diversity of dimensions not only between different studies but also among individuals in the same study sample, as well as between different sites in the same individual (Table 2). This should not be surprising, as every anatomic measurement in humans comes with a physiological range, often representing a normal distribution. Therefore, the quest for defining a single number for the proper vertical dimensions of the Connective Tissue and the Junctional Epithelium around implants is probably futile and misguided. On the other hand, if we exclude a few outliers in the available human studies, the range of 2.5–4 mm appears as a “comfort zone,” representing the essential height for the establishment and maintenance of the peri‐implant tissue seal in healthy conditions. This resembles the concept of the “Biologic width,” which was first introduced around natural teeth and represented the essential supracrestal height of the periodontal tissue. If violated by restorations or trauma, marginal bone loss would follow. Today, there is significant evidence pointing to a similar concept around implants (Linkevicius, Puisys, Linkevieiene, et al., 2015; Linkevicius, Puisys, Steigmann, et al., 2015); thus, it becomes important to establish a minimum peri‐implant supracrestal vertical tissue height of about 3 to 4 mm, which will accommodate adequately the biologic demands of sustainable health. In cases where the vertical height of the peri‐implant tissue is less than 3 mm, marginal bone resorption has often been reported around the implant platform (Puisys & Linkevicius, 2015). This might be physiological remodeling, resulting in re‐establishing the vertical dimensions required to accommodate the soft tissues at the expense of the crestal peri‐implant bone.

**Table 2:** Vertical height of peri‐implant tissue in human histomorphometric studies as organized in sulcus, junctional epithelium, connective tissue, and total mean in mm and (standard deviation).

Author/year		Sulcus	JE	CT	Total
Glauser et al., 2005		0.5 (0.1)	2.9 (0.7)	0.7 (0.2)	4.1 (1.3)
Tomasi et al., 2014		‐	1.6 (0.6)	0.8 (0.7)	2.4 (0.7)
Romanos et al., 2010	Maxilla	2.7 (0.8)	1.3 (0.4)	2.5 (1.3)	6.5 (2.5)
	Mandible	1.7 (0.4)	1.5 (0.5)	1.6 (0.4)	4.8 (1.3)

## 3 OVERCONTOURING OF THE PROSTHESIS, EMERGENCE ANGLE, AND CLINICAL IMPLICATIONS

“Overcontouring”—an excessively wide profile of the abutment or prosthesis—has been associated with two main unfavorable clinical outcomes: early “aseptic” marginal bone loss or “remodeling” and peri‐implantitis. The pathogenetic mechanisms involved in the two can be very different. Histomorphometry on animals showed that a healing abutment with a 45° angle led to significantly more marginal bone loss than the equivalent with 15° after only 4 months of healing (Souza et al., 2018). This angle corresponds to a steep widening of the abutment diameter right coronal of the implant platform and in close proximity to the marginal bone. At the same time, cross‐sectional studies (Katafuchi et al., 2018; Majzoub et al., 2021; Yi et al., 2020) have suggested that overcontouring of the prosthesis more than 30° as it appears in peri‐apical radiographs is correlated with an increased risk for peri‐implantitis in bone‐level implants. Finally, the convexity of the prosthesis profile has been correlated with increased recession (Siegenthaler et al., 2022), marginal bone loss (Valente et al., 2020), and when combined with overcontouring, peri‐implantitis (Yi et al., 2020). Collectively seen, these findings appear to relate to the previously discussed concepts of vertical tissue height and biologic width, as an excessively wide angle of ascendance from the bone margin can also be seen as limiting the essential vertical space for the establishment of the soft tissue seal.

## 4 DESIGNING FAVORABLE DIMENSIONS FOR THE IMPLANT SUPRACRESTAL COMPLEX

**Figure 1:**
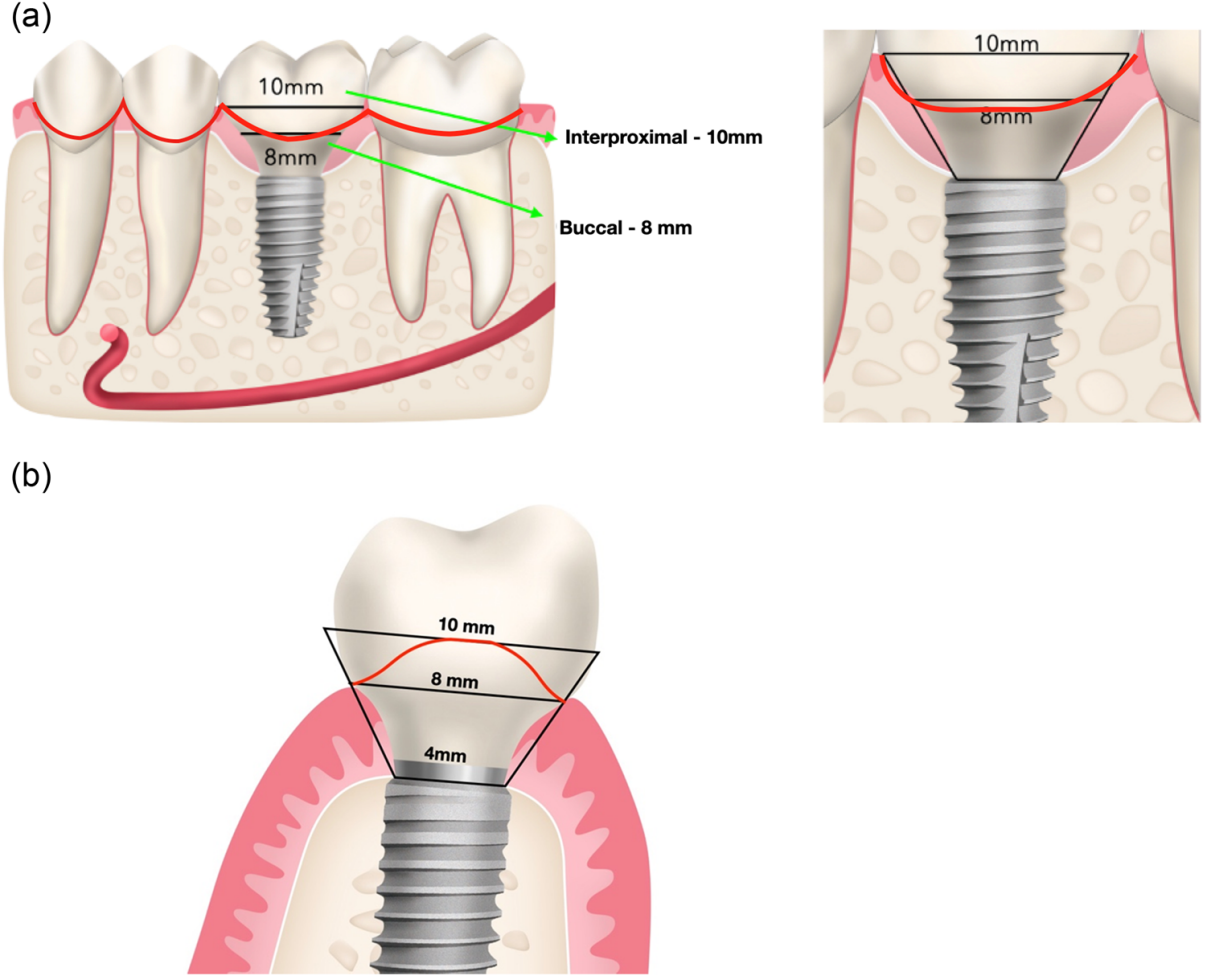
Approximation of the implant supracrestal complex with a trapezoid rectangle in the case of a posterior tooth (a) from a buccolingual and (b) mesiodistal perspective.

In clinical practice, overcontouring is not uncommon, and in most cases encountered is a compensation for deficiencies in the implant position. In particular, possibly the most frequent reason for overcontouring is the inability to secure an adequate vertical height between the implant platform and the margin of the prosthesis. The “contour” of the transmucosal components of the implant is what determines the transition from the narrow and cylindrical implant platform to the wider and rectangular or oval‐shaped cervical margin of the crown. For this transition to be smooth and gradual, an adequate vertical height is required; otherwise, the contour will present a steep transition with wide contour angles. This transition can be approached with a simple mathematical equation. We can approximate the transmucosal components of the implant and prosthesis with a trapezoid rectangle. In the case of a posterior implant prosthesis, the implant supracrestal complex represents a transition from a commonly 4 mm wide implant platform to a cervical margin with an approximate width of 8 mm at the buccal/lingual and 10 mm at the interproximal area (Figure 1a,b).

**Figure 2:**
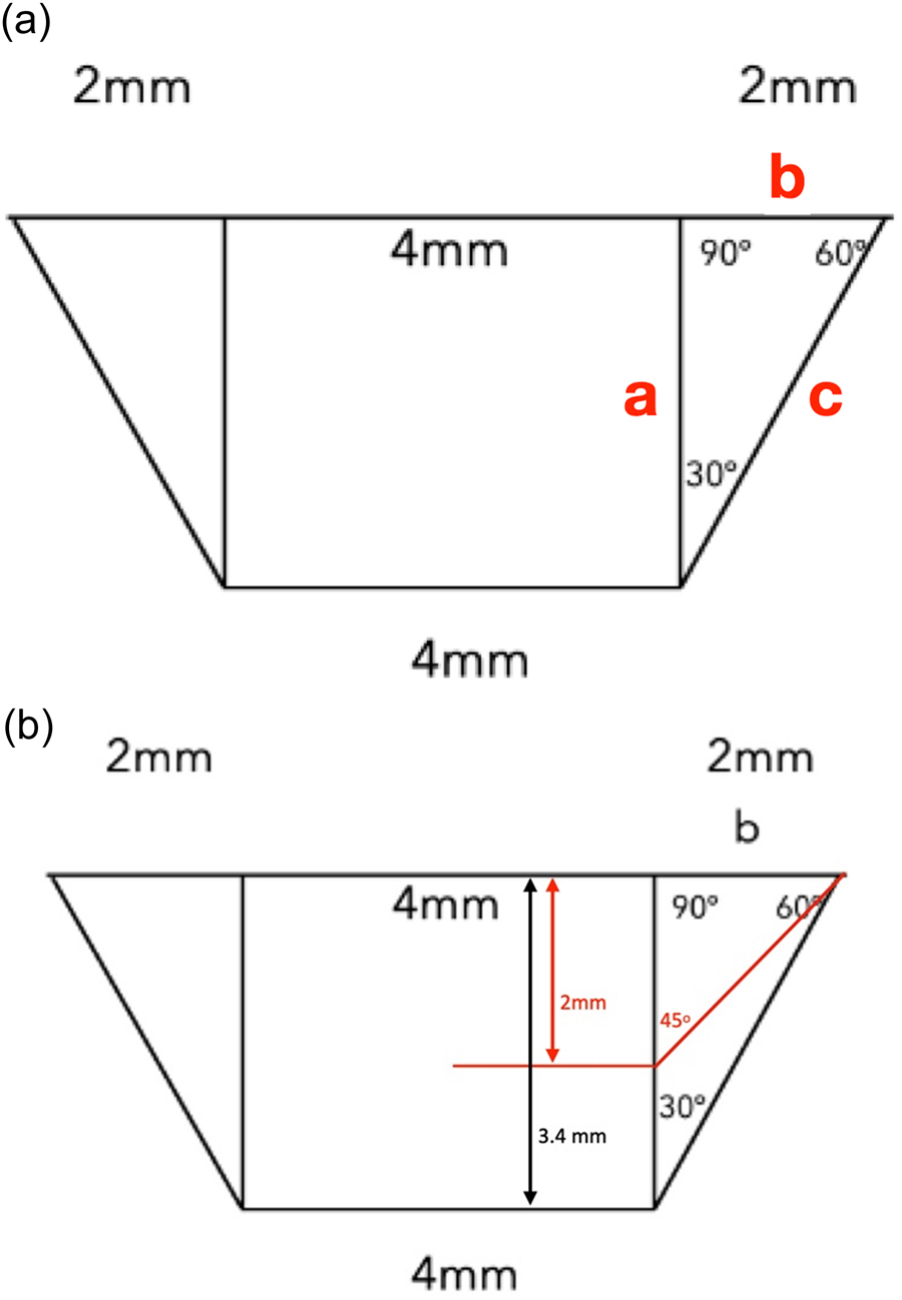
Application of the Pythagorean theorem in the trapezoid approximation of the Implant Supracrestal Complex for a posterior tooth (a) calculation of essential height for a 30° contour angle (b) increase of the angle for height reduction to 2 mm (red line).

If the angle of the contour is to be 30°, the Pythagoras theorem can be utilized to calculate the corresponding essential height, where a^2^=b^2^+c^2^ (Figure 2a). With a simple calculation, we can reach the conclusion that for the above configuration, an essential height of 3.4 mm is required between the implant platform and the buccal mucosal margin, while 5.1 mm is required between the platform and the papilla area. On the contrary, starting with a compromised vertical height can only lead to a steeper transition through a wider contour angle. Reducing the height to only 2 mm in the above example (Figure 2b) would result in a drastic increase of the contour angle to 45°.

## 5 CLINICAL IMPLICATIONS

**Figure 3:**
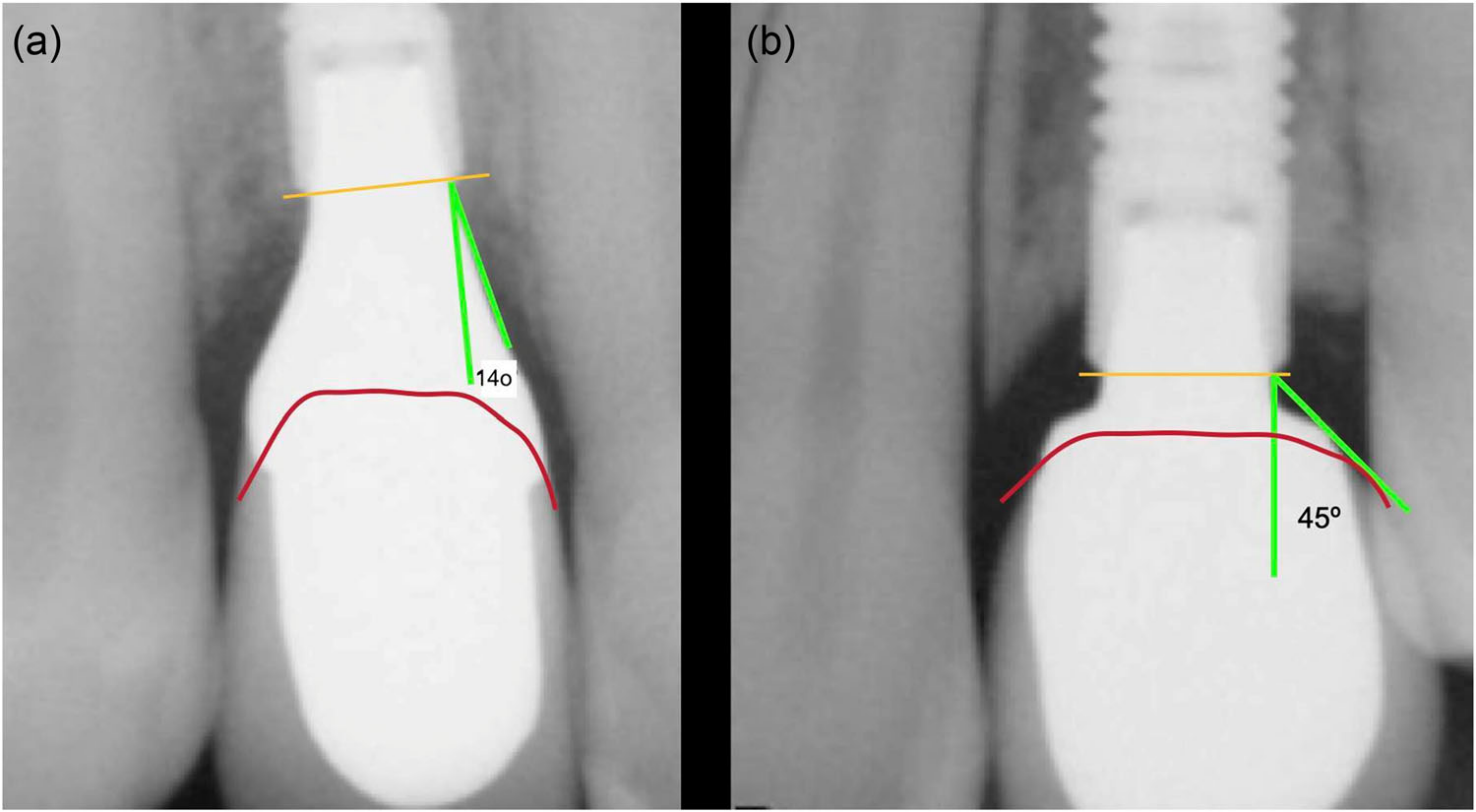
Failure to secure the proper vertical dimension of the implant supracrestal complex will result in a wide contour angle and could be associated with marginal bone loss. (a‐left) adequate vertical dimension allows for a narrow contour angle. (b‐right) Deficient vertical dimension or “shallow” placement in relation to the prosthetic cervical margin resulting in a wide contour angle and possibly related to the observed marginal bone loss.

Clinical experience, as discussed above, suggests that the vertical dimension of the Implant Supracrestal complex and the contour angle of the transmucosal components are closely interrelated. Consequently, if we accept that a contour angle of no more than 30° is desired (Katafuchi et al., 2018; Yi et al., 2020), we must similarly accept the need for an appropriate circumferential minimum vertical dimension between 3 and 4 mm. This vertical dimension represents the distance between the implant platform at the bone margin and the cervical contour of the prosthesis (Figure 3) and is not to be confused with the available height of the soft tissue. The cervical margin of the prosthesis is determined by the natural shape of the crown of the tooth to be replaced and is directed by the corresponding shape and size of neighboring teeth, smile line, and esthetic needs (Buser et al., 2004). The diagnostic digital wax‐up will define the optimal cervical margin of the prosthesis based on the neighboring teeth, the physiological esthetics and local anatomic conditions. Consequently, clinicians have to assess carefully the anatomic landmarks in the digital treatment planning in a “top to bottom” sequence: the cervical margin of the implant crown at buccal and interproximal areas is what will then dictate the respective minimum vertical distance to the implant platform at each area. A vertical distance of less than 3 mm at any point will result in an unfavorable contour angle (Figure 3). Increasing the thickness of the soft tissue will not have any meaning in such a case since the cervical margin of the prosthesis is directed by the esthetics and the neighboring teeth and has to be in the same place regardless of the thickness of the soft tissue. In such cases, the only way to create the essential vertical dimension and the favorable contour angle is to place the implant subcrestally. Subcrestal implant placement might therefore be essential in the case of a short vertical height to establish a vertical height of at least 3 mm to the cervical margin of the prosthesis. Souza et al. (2018) described the presence of a “biological compartment” when implants are placed subcrestally, which can be an element of tissue stability. To not violate this compartment, it is important to follow an angle as close to 0° as possible for the subcrestal part of the abutment and only widen toward the final contour when coronal of the bone margin. In other words, in the case of subcrestal placement, the shape of the subcrestal component of the abutment should ideally be cylindrical until clearing the marginal bone and then following the desirable angulation to reach the cervical margin.

The calculation of the contour is equally valid for tissue‐ or bone‐level implants. From a biological point of view, there exists an endosseous, a transmucosal, and an oral component in any implant prosthesis. In tissue‐level (TL) implants, the endosseous and a part of the transmucosal components are interlocked in the same mechanical unit (TL implant), while the same biological area corresponds to two mechanical units (implant‐abutment) in the case of bone‐level (BL) design. From the clinicians' point of view, the difference with tissue‐level implants is that the design of the most apical 2–3 mm of the transmucosal component is “predetermined,” as this is directed by the collar of the implant and cannot be altered or customized. There exist diverse designs of tissue‐level implants with regard to the height of the transmucosal collar, as well as its contour angle. However, in most tissue‐level implants, the contour angle of the collar will not exceed 30°. This fact, when combined with a collar height close to 3 mm, allows the tissue‐level implants to act as a “pre‐programmed” design, preventing unfavorable designs and overcontouring from the bone‐level. At the same time, as discussed above, tissue‐level implants are not indicated for subcrestal placement, while the polished collar should stay coronal of the bone (Buser et al., 2004), limiting the designers' ability to make adjustments to the vertical dimension when required.

The principles discussed in this paper should be seen through the limitations of the clinical realities. The Implant Supracrestal Complex is a 3‐dimensional structure with complex geometry as well as frequent interchanges between concavity and convexity. Approaching it through a 2‐dimensional geometry might not always correspond to the clinical challenges. Nevertheless, digital technology and Computer Assisted Design can now empower the designer to visualize and design the Implant Supracrestal Complex from every angle, converting the 2‐dimensional design principles into a proper fully 3‐dimensional structure.

A simple geometric equation can clearly illustrate the interrelation between vertical height and the contour angle of the transmucosal component of the implant prosthesis. This can contribute to the correct assessment of the essential circumferential soft tissue height required for a specific implant and prosthesis diameter and thus identify with precision the optimal implant position. Furthermore, this equation could have future applications in CAD and algorithmic programs that support treatment planning and manufacturing of implant components.

The interrelation of vertical height and contour angle, as discussed in this paper, is based on clinical observation and simple geometrical calculations. Future research will be required to investigate the interrelation of tissue dimensions and prosthesis design, as well as possible associations with clinical outcomes in large samples of patients. With the possibilities introduced by 3‐dimensional imaging, future research could assess with precision the soft and hard peri‐implant tissue dimensions, calculate contour angles of the prosthesis, and investigate associations with clinical outcomes.

## AUTHOR CONTRIBUTIONS

**Algirdas Puisys**: Conceptualization; data curation; formal analysis; investigation; methodology; project administration; resources; supervision; validation; visualization; writing—review & editing. **Martin Janda**: Conceptualization; investigation; methodology; validation; writing—review & editing. **German O. Gallucci**: Conceptualization; methodology; validation; writing—review & editing. **Nikos Mattheos**: Conceptualization; data curation; formal analysis; funding acquisition; investigation; methodology; project administration; supervision; validation; visualization; Writing—original draft; writing—review & editing. **Viktorija Auzbikaviciute**: Data curation; resources; visualization; writing—review & editing.

## CONFLICT OF INTEREST STATEMENT

The authors declare no conflict of interest.

## Data Availability

Data are available from the corresponding author upon reasonable request.
